# Supplementary material for: A Likelihood Approach for Real-Time Calibration of Stochastic Compartmental Epidemic Models
Source: PLoS Comput Biol. 2017 Jan 17;13(1):e1005257. doi: 10.1371/journal.pcbi.1005257 (PMC5240920; doi:10.1371/journal.pcbi.1005257)
Supplement: S1 File — (TAR.GZ) [file pcbi.1005257.s014.tar.gz › HSPH_Online-SI-Revision/MSS10/n100-extreme/n100-extreme_NEW2_table-4.pdf]

1	newly 3 weeks spec	10683.	9263.39	{8700., 9883.}	13.418	0.0631611	newly 3 weeks cumul	25079.	23058.6	{21484., 24782.}	8.23219	0.0379026	newly 5 weeks spec	10855.	6464.6	{6045., 6662.}	40.4488	0.227489	newly 5 weeks cumul	47373.	38013.4	{36273., 39585.}	19.8043	0.0964179	newly 58 weeks cumul	85292.	48988.6	{46960., 49265.}	42.5637	0.243042
2	newly 3 weeks spec	8383.	4682.27	{4178., 5141.}	44.1457	0.254795	newly 3 weeks cumul	21608.	15435.4	{13458., 17093.}	28.5661	0.147777	newly 5 weeks spec	8027.	2405.18	{2034., 2640.}	70.0364	0.529853	newly 5 weeks cumul	38163.	21361.4	{19322., 22791.}	44.0259	0.253511	newly 54 weeks cumul	72415.	25154.4	{23288., 26114.}	65.2635	0.461585
3	newly 3 weeks spec	12915.	10470.6	{9958., 10852.}	18.9271	0.0915565	newly 3 weeks cumul	29820.	25607.	{24201., 27219.}	14.1281	0.0666466	newly 5 weeks spec	11421.	6616.94	{6213., 6942.}	42.0634	0.238012	newly 5 weeks cumul	54424.	41473.5	{39763., 42611.}	23.7956	0.118337	newly 59 weeks cumul	84212.	50801.2	{49775., 51352.}	39.6746	0.219831
4	newly 3 weeks spec	15590.	16430.5	{15834., 17377.}	5.40244	0.0225323	newly 3 weeks cumul	27674.	29104.6	{27732., 31021.}	5.24792	0.0217933	newly 5 weeks spec	15345.	15481.3	{15083., 15886.}	2.66849	0.0117478	newly 5 weeks cumul	61225.	63382.7	{62266., 65156.}	3.55303	0.0150961	newly 63 weeks cumul	88766.	90994.6	{91561., 92062.}	3.90445	0.0168969
5	newly 3 weeks spec	10521.	9832.15	{9185., 11103.}	9.28191	0.0421208	newly 3 weeks cumul	21016.	19929.9	{18594., 22594.}	8.53883	0.0383017	newly 5 weeks spec	12782.	11882.	{11170., 12298.}	7.04154	0.0323395	newly 5 weeks cumul	46785.	43711.1	{42216., 46329.}	6.87148	0.0311139	newly 60 weeks cumul	85329.	82435.1	{67487., 88322.}	8.28962	0.0392119
6	newly 3 weeks spec	10702.	10577.2	{10252., 10939.}	2.37713	0.0104949	newly 3 weeks cumul	23963.	23647.7	{22711., 24680.}	2.76969	0.0122062	newly 5 weeks spec	11271.	10580.9	{10291., 11171.}	6.25428	0.0285827	newly 5 weeks cumul	47247.	45749.5	{44490., 47532.}	3.48179	0.0155512	newly 58 weeks cumul	86171.	74757.5	{71847., 88114.}	14.0505	0.0668948
7	newly 3 weeks spec	11555.	10309.7	{9611., 10855.}	10.8912	0.0505082	newly 3 weeks cumul	26529.	25503.7	{23750., 27285.}	5.38848	0.024237	newly 5 weeks spec	11237.	6562.09	{6088., 6862.}	41.6028	0.2352	newly 5 weeks cumul	49918.	41156.5	{39131., 42784.}	17.5518	0.0842254	newly 56 weeks cumul	84780.	50747.7	{49340., 51283.}	40.1419	0.223474
8	newly 3 weeks spec	11018.	9628.17	{8889., 10464.}	12.6142	0.059407	newly 3 weeks cumul	24447.	22196.	{20737., 23615.}	9.24277	0.0427706	newly 5 weeks spec	11207.	8470.43	{6726., 10553.}	24.4184	0.130322	newly 5 weeks cumul	47781.	40551.8	{36974., 44925.}	15.1298	0.0727266	newly 58 weeks cumul	87124.	59779.3	{48516., 73595.}	31.4121	0.172327
9	newly 3 weeks spec	10870.	9099.74	{8101., 9990.}	16.3091	0.0786076	newly 3 weeks cumul	22125.	19293.1	{17005., 21388.}	13.2397	0.0629007	newly 5 weeks spec	12694.	8582.57	{8012., 9011.}	32.3888	0.170611	newly 5 weeks cumul	47679.	37818.7	{35165., 40371.}	20.6806	0.101265	newly 58 weeks cumul	88989.	53135.6	{51106., 53949.}	40.2897	0.224342
10	newly 3 weeks spec	15442.	13524.1	{12796., 14220.}	12.4201	0.0580498	newly 3 weeks cumul	29673.	26118.7	{24418., 27861.}	11.9781	0.0559531	newly 5 weeks spec	11765.	13620.9	{12104., 14208.}	16.0681	0.0641726	newly 5 weeks cumul	57244.	55291.2	{51885., 57465.}	3.67874	0.016602	newly 62 weeks cumul	72720.	83040.	{71068., 89882.}	15.38	0.0615657
11	newly 3 weeks spec	9197.	5192.56	{4604., 5716.}	43.5407	0.25016	newly 3 weeks cumul	23145.	16551.5	{14145., 18715.}	28.4879	0.147953	newly 5 weeks spec	8893.	2622.78	{2305., 2861.}	70.5074	0.532568	newly 5 weeks cumul	41559.	23076.2	{20812., 25124.}	44.4737	0.256937	newly 56 weeks cumul	75767.	27039.	{25406., 28562.}	64.3129	0.44838
12	newly 3 weeks spec	11654.	11721.1	{11075., 12216.}	3.77836	0.0165946	newly 3 weeks cumul	24296.	24795.	{23345., 25956.}	4.46777	0.0192837	newly 5 weeks spec	12745.	10376.6	{10083., 10834.}	18.5831	0.0902742	newly 5 weeks cumul	50388.	47626.2	{45026., 49129.}	5.50196	0.0250798	newly 57 weeks cumul	88506.	65888.2	{65877., 67515.}	25.5551	0.128924
13	newly 3 weeks spec	8371.	8142.75	{7844., 8403.}	2.99689	0.0133157	newly 3 weeks cumul	19985.	19733.6	{19105., 20350.}	2.12029	0.00931988	newly 5 weeks spec	8948.	8240.04	{8072., 8438.}	7.91194	0.035981	newly 5 weeks cumul	38014.	36631.4	{35829., 37498.}	3.64584	0.0162205	newly 55 weeks cumul	83218.	64174.9	{63814., 64746.}	22.8834	0.113
14	newly 3 weeks spec	11434.	13455.3	{8894., 16311.}	28.6867	0.124271	newly 3 weeks cumul	18644.	23904.8	{13366., 32498.}	41.1375	0.164462	newly 5 weeks spec	15310.	7795.12	{4322., 12189.}	49.0963	0.325162	newly 5 weeks cumul	50732.	44503.5	{39882., 47554.}	12.874	0.0633617	newly 60 weeks cumul	70240.	50472.9	{48495., 53060.}	28.1422	0.144432
15	newly 3 weeks spec	13412.	12253.7	{11206., 12994.}	8.67216	0.0400877	newly 3 weeks cumul	27516.	25278.3	{23765., 26780.}	8.21064	0.0378704	newly 5 weeks spec	12056.	11497.	{10304., 12880.}	9.714	0.0446086	newly 5 weeks cumul	54113.	50155.5	{46152., 53410.}	7.31334	0.0337984	newly 60 weeks cumul	75951.	72772.	{66616., 81531.}	10.9666	0.0498873
16	newly 3 weeks spec	15726.	15736.9	{14696., 18011.}	5.89253	0.0253172	newly 3 weeks cumul	30526.	30580.8	{28200., 34387.}	5.8156	0.0249493	newly 5 weeks spec	14011.	12344.7	{11896., 13087.}	11.9097	0.0586416	newly 5 weeks cumul	61514.	59209.	{56902., 64744.}	5.8487	0.0265218	newly 60 weeks cumul	88752.	76592.3	{76274., 80900.}	13.7008	0.0656452
17	newly 3 weeks spec	9053.	5279.59	{4761., 5847.}	41.6813	0.235797	newly 3 weeks cumul	21971.	15425.7	{13230., 17653.}	29.7906	0.15637	newly 5 weeks spec	9419.	3075.71	{2754., 3431.}	67.3457	0.487583	newly 5 weeks cumul	40887.	22789.8	{20432., 25271.}	44.2616	0.255182	newly 55 weeks cumul	79924.	27732.7	{26000., 29293.}	65.3012	0.460098
18	newly 3 weeks spec	8959.	8741.69	{8515., 8975.}	2.76448	0.0122296	newly 3 weeks cumul	19558.	19357.7	{18823., 19876.}	1.7872	0.00783907	newly 5 weeks spec	10392.	10077.1	{9847., 10301.}	3.05504	0.0135452	newly 5 weeks cumul	40376.	39548.8	{38864., 40293.}	2.17761	0.00959302	newly 56 weeks cumul	75084.	72595.8	{72516., 73651.}	3.31388	0.0148058
19	newly 3 weeks spec	11412.	11693.	{11111., 12306.}	4.18647	0.0177819	newly 3 weeks cumul	23492.	24507.4	{23079., 25968.}	5.10557	0.0213731	newly 5 weeks spec	12862.	10588.8	{10031., 11015.}	17.6742	0.085785	newly 5 weeks cumul	49674.	47654.	{45659., 49733.}	4.45026	0.0200401	newly 58 weeks cumul	89565.	66866.	{66225., 67800.}	25.3436	0.128213
20	newly 3 weeks spec	15824.	10761.5	{7052., 14058.}	32.3665	0.186268	newly 3 weeks cumul	21990.	15719.2	{10257., 21332.}	29.2322	0.167989	newly 5 weeks spec	20039.	17533.6	{14110., 19905.}	13.1151	0.0639575	newly 5 weeks cumul	66304.	51546.6	{43147., 58196.}	22.364	0.11454	newly 63 weeks cumul	91232.	72506.9	{66142., 78081.}	20.5247	0.102299
21	newly 3 weeks spec	11565.	11822.5	{10711., 12666.}	5.90445	0.0253133	newly 3 weeks cumul	23774.	24505.	{22555., 26546.}	6.26634	0.0266822	newly 5 weeks spec	12361.	10388.7	{8731., 11160.}	16.1275	0.0783882	newly 5 weeks cumul	49979.	47516.1	{43156., 50496.}	5.44625	0.0250128	newly 60 weeks cumul	80337.	64728.	{56486., 67853.}	19.6272	0.0963509
22	newly 3 weeks spec	13043.	12364.8	{11237., 13124.}	5.60117	0.025876	newly 3 weeks cumul	25673.	25040.4	{23221., 26476.}	4.23951	0.0192656	newly 5 weeks spec	13126.	12286.	{9131., 13595.}	9.83917	0.0486966	newly 5 weeks cumul	53143.	51191.4	{44102., 54766.}	5.62178	0.0260705	newly 61 weeks cumul	81637.	78996.4	{57215., 88365.}	12.9636	0.0613257
23	newly 3 weeks spec	8971.	7362.56	{6894., 7849.}	17.9293	0.0879626	newly 3 weeks cumul	22691.	20130.5	{18847., 21326.}	11.2843	0.0528502	newly 5 weeks spec	8717.	5484.91	{4797., 5939.}	37.078	0.209004	newly 5 weeks cumul	40575.	32361.5	{30387., 34370.}	20.2428	0.100145	newly 54 weeks cumul	74886.	44823.6	{39671., 47338.}	40.1442	0.226913
24	newly 3 weeks spec	9753.	6064.39	{5223., 6589.}	37.8203	0.21047	newly 3 weeks cumul	22644.	16677.8	{13874., 19065.}	26.4688	0.137472	newly 5 weeks spec	10377.	3736.22	{3208., 4133.}	63.9952	0.450439	newly 5 weeks cumul	43672.	25557.6	{22652., 27447.}	41.4783	0.236068	newly 54 weeks cumul	86096.	31544.	{29279., 32112.}	63.3619	0.439186
25	newly 3 weeks spec	14475.	11185.5	{9787., 12617.}	22.7254	0.113988	newly 3 weeks cumul	28620.	23349.1	{20181., 26163.}	18.8968	0.0929421	newly 5 weeks spec	13481.	8589.63	{6674., 9585.}	36.2834	0.200274	newly 5 weeks cumul	57956.	43195.1	{39139., 45675.}	25.4691	0.128561	newly 57 weeks cumul	86463.	55093.	{46740., 57781.}	36.2814	0.196892
26	newly 3 weeks spec	8715.	6367.74	{5000., 8568.}	27.083	0.145025	newly 3 weeks cumul	21621.	17785.9	{14812., 21424.}	17.948	0.0893488	newly 5 weeks spec	8698.	4480.46	{2528., 8199.}	48.4886	0.327707	newly 5 weeks cumul	39423.	27893.1	{21751., 38747.}	29.2716	0.159351	newly 55 weeks cumul	81661.	38228.6	{26816., 65987.}	53.1862	0.353488
27	newly 3 weeks spec	9364.	8993.46	{8765., 9254.}	4.00897	0.0178686	newly 3 weeks cumul	24919.	24785.7	{24164., 25505.}	1.64252	0.00717521	newly 5 weeks spec	8776.	6652.3	{6452., 6844.}	24.199	0.120432	newly 5 weeks cumul	43318.	39615.1	{38968., 40359.}	8.5482	0.0388581	newly 56 weeks cumul	79621.	55376.8	{54815., 55934.}	30.4495	0.157714
28	newly 3 weeks spec	11755.	9664.75	{8972., 10375.}	17.7818	0.0859395	newly 3 weeks cumul	27332.	23875.5	{22017., 26041.}	12.6465	0.0596683	newly 5 weeks spec	11093.	6543.99	{6193., 6893.}	41.0079	0.230067	newly 5 weeks cumul	50746.	39173.9	{37287., 41435.}	22.8039	0.112995	newly 60 weeks cumul	87654.	49285.	{47964., 50800.}	43.7732	0.250451
29	newly 3 weeks spec	8104.	5977.77	{4873., 6943.}	26.2368	0.136827	newly 3 weeks cumul	20099.	16624.5	{14332., 18360.}	17.2869	0.0849804	newly 5 weeks spec	8778.	4233.6	{2739., 5735.}	51.7703	0.338756	newly 5 weeks cumul	37699.	26196.8	{21598., 30414.}	30.5106	0.162655	newly 54 weeks cumul	75236.	35379.1	{26619., 44165.}	52.9758	0.338933
30	newly 3 weeks spec	13462.	15379.	{13194., 18025.}	16.678	0.0660474	newly 3 weeks cumul	25138.	30062.5	{24603., 35375.}	22.7974	0.0885242	newly 5 weeks spec	14527.	7849.66	{3706., 11211.}	46.3319	0.306942	newly 5 weeks cumul	55394.	51146.2	{44357., 59146.}	12.1626	0.0570468	newly 63 weeks cumul	91285.	58654.2	{47558., 69262.}	35.7783	0.200273
31	newly 3 weeks spec	13467.	14898.	{12978., 16941.}	12.7297	0.0510075	newly 3 weeks cumul	21982.	24234.8	{20737., 28004.}	13.2266	0.0529862	newly 5 weeks spec	17282.	15487.3	{12572., 17437.}	10.8941	0.0565019	newly 5 weeks cumul	58272.	58741.6	{55555., 62936.}	4.77166	0.020882	newly 64 weeks cumul	87865.	79697.8	{66235., 92466.}	11.2171	0.0538105
32	newly 3 weeks spec	11691.	11279.4	{11022., 11535.}	3.52066	0.015625	newly 3 weeks cumul	23586.	23070.3	{22499., 23597.}	2.36907	0.0104564	newly 5 weeks spec	12032.	12821.4	{12674., 12986.}	6.56109	0.0275788	newly 5 weeks cumul	48919.	49172.7	{48517., 49786.}	0.890411	0.00384828	newly 61 weeks cumul	74173.	87590.7	{87387., 87753.}	18.0897	0.0722093
33	newly 3 weeks spec	9366.	9845.37	{9392., 10325.}	6.18952	0.0260977	newly 3 weeks cumul	20595.	21762.5	{20807., 22639.}	6.19835	0.0260325	newly 5 weeks spec	10722.	10205.2	{7451., 11066.}	6.33744	0.0310911	newly 5 weeks cumul	42102.	42873.9	{38054., 45016.}	4.90578	0.0214249	newly 59 weeks cumul	87634.	70549.	{50332., 74967.}	19.4958	0.0978353
34	newly 3 weeks spec	6522.	4846.58	{4295., 5332.}	25.6887	0.130765	newly 3 weeks cumul	16514.	13442.2	{11868., 14627.}	18.6014	0.0906481	newly 5 weeks spec	7135.	3920.63	{3209., 4731.}	45.0507	0.264787	newly 5 weeks cumul	30872.	21927.7	{19183., 24296.}	28.9722	0.150282	newly 54 weeks cumul	67088.	32701.1	{27320., 38718.}	51.2563	0.315511
35	newly 3 weeks spec	10120.	10159.2	{9870., 10450.}	1.92717	0.00834472	newly 3 weeks cumul	20852.	20531.3	{19986., 21119.}	2.23302	0.00983829	newly 5 weeks spec	12158.	12557.1	{12297., 12790.}	3.29479	0.0140361	newly 5 weeks cumul	45344.	45591.9	{44685., 46410.}	1.23306	0.00532832	newly 59 weeks cumul	90553.	84522.6	{83852., 85304.}	6.66237	0.0300419
36	newly 3 weeks spec	8518.	5163.18	{4546., 5644.}	39.3851	0.218912	newly 3 weeks cumul	20967.	15619.1	{13429., 17486.}	25.5062	0.130124	newly 5 weeks spec	8858.	2878.17	{2584., 3197.}	67.5077	0.489684	newly 5 weeks cumul	39044.	22616.4	{20385., 24505.}	42.0745	0.238183	newly 57 weeks cumul	75101.	27234.2	{26011., 28660.}	63.7365	0.440851
37	newly 3 weeks spec	14436.	15140.3	{13632., 15907.}	8.11056	0.034779	newly 3 weeks cumul	27190.	29144.9	{27716., 30846.}	9.7178	0.0409043	newly 5 weeks spec	14344.	13095.7	{8849., 14482.}	9.18293	0.0506622	newly 5 weeks cumul	57920.	58487.3	{46116., 61925.}	6.62768	0.0299936	newly 62 weeks cumul	85971.	81077.	{55117., 89349.}	8.66482	0.0435489
38	newly 3 weeks spec	14671.	13242.3	{8062., 16555.}	17.5669	0.0919074	newly 3 weeks cumul	23153.	22759.8	{12341., 31079.}	25.8989	0.124867	newly 5 weeks spec	16782.	8675.72	{4925., 13208.}	48.3456	0.318119	newly 5 weeks cumul	59731.	45074.4	{39535., 49191.}	24.5377	0.12462	newly 62 weeks cumul	80717.	51708.	{48528., 54145.}	35.9392	0.195202
39	newly 3 weeks spec	8107.	6632.43	{5224., 8764.}	21.9905	0.112723	newly 3 weeks cumul	18981.	17125.1	{13607., 20360.}	13.2094	0.0640501	newly 5 weeks spec	8590.	5451.37	{3291., 9655.}	42.4348	0.270103	newly 5 weeks cumul	36334.	28964.1	{22267., 39822.}	24.7781	0.129156	newly 56 weeks cumul	64192.	43775.3	{29069., 76104.}	40.3804	0.240976
40	newly 3 weeks spec	9375.	5179.2	{4650., 5728.}	44.7552	0.258865	newly 3 weeks cumul	23924.	16722.4	{14805., 18570.}	30.1021	0.157075	newly 5 weeks spec	8935.	2504.56	{2255., 2756.}	71.9691	0.553758	newly 5 weeks cumul	42535.	23020.7	{20959., 24744.}	45.8782	0.267446	newly 57 weeks cumul	74746.	26661.4	{24952., 28215.}	64.3306	0.448117
41	newly 3 weeks spec	9002.	5848.4	{5094., 6273.}	35.5679	0.194491	newly 3 weeks cumul	21615.	17008.7	{14626., 19480.}	21.9094	0.110095	newly 5 weeks spec	8585.	3414.81	{2657., 3407.}	61.4056	0.428958	newly 5 weeks cumul	39635.	25172.7	{22011., 26823.}	37.2007	0.205724	newly 58 weeks cumul	64657.	31448.9	{27247., 30546.}	53.1574	0.335437
42	newly 3 weeks spec	13145.	14006.4	{13103., 15372.}	7.22199	0.0299117	newly 3 weeks cumul	25907.	28876.5	{26870., 31475.}	11.9094	0.0478669	newly 5 weeks spec	11817.	10178.4	{5551., 13902.}	28.1863	0.157272	newly 5 weeks cumul	52038.	52637.6	{45472., 58457.}	10.2956	0.0446543	newly 62 weeks cumul	70332.	70041.5	{50392., 89464.}	23.0288	0.103091
43	newly 3 weeks spec	10255.	9104.47	{8378., 9981.}	11.6602	0.0551864	newly 3 weeks cumul	22579.	20985.8	{19116., 22990.}	7.97861	0.037179	newly 5 weeks spec	11209.	7557.68	{6892., 9758.}	32.5749	0.175252	newly 5 weeks cumul	45260.	37678.5	{35290., 41876.}	16.8269	0.0815934	newly 55 weeks cumul	87509.	51727.1	{48451., 65228.}	40.8894	0.232202
44	newly 3 weeks spec	15502.	15011.6	{14683., 15365.}	3.26409	0.0145001	newly 3 weeks cumul	33206.	31950.8	{31104., 32863.}	3.79642	0.0169025	newly 5 weeks spec	12451.	12471.4	{10536., 12951.}	4.39459	0.0198177	newly 5 weeks cumul	61309.	59617.2	{56339., 60670.}	2.77657	0.0123929	newly 61 weeks cumul	87960.	85966.6	{70111., 88604.}	3.17023	0.0150057
45	newly 3 weeks spec	16177.	13521.1	{12348., 14961.}	16.7462	0.0809653	newly 3 weeks cumul	31598.	27765.9	{23659., 31948.}	13.5449	0.0654847	newly 5 weeks spec	13010.	7815.26	{5584., 11894.}	40.3365	0.241428	newly 5 weeks cumul	61499.	47771.9	{43655., 58018.}	22.3208	0.111682	newly 60 weeks cumul	80329.	55759.	{50301., 76185.}	30.7868	0.164127
46	newly 3 weeks spec	10769.	11248.1	{10854., 11623.}	4.97103	0.0210387	newly 3 weeks cumul	20202.	21384.9	{20600., 22028.}	6.36219	0.026738	newly 5 weeks spec	14108.	13968.2	{13800., 14213.}	1.42926	0.00650099	newly 5 weeks cumul	48516.	49516.2	{48773., 50415.}	2.85452	0.0123295	newly 60 weeks cumul	82843.	89105.4	{88552., 90845.}	9.4776	0.0400811
47	newly 3 weeks spec	12425.	10945.9	{9492., 12053.}	12.0095	0.0570996	newly 3 weeks cumul	24055.	22181.1	{19132., 24439.}	8.61027	0.0404228	newly 5 weeks spec	13477.	10007.7	{8829., 11597.}	25.7422	0.131622	newly 5 weeks cumul	52391.	44197.7	{39351., 49019.}	15.6388	0.0752461	newly 62 weeks cumul	79732.	60113.	{53853., 68136.}	24.6062	0.124357
48	newly 3 weeks spec	12536.	11516.6	{10376., 12384.}	10.3821	0.0475218	newly 3 weeks cumul	21158.	19548.7	{17450., 21431.}	9.99754	0.0457421	newly 5 weeks spec	16548.	15727.8	{15229., 16263.}	5.02254	0.0226718	newly 5 weeks cumul	54873.	51363.2	{48800., 53725.}	7.19048	0.0325017	newly 62 weeks cumul	90499.	83053.5	{77797., 92747.}	9.31327	0.0431579
49	newly 3 weeks spec	21183.	15674.7	{13000., 18415.}	26.0035	0.139709	newly 3 weeks cumul	34718.	25634.6	{19983., 31162.}	26.1635	0.143811	newly 5 weeks spec	14957.	12528.6	{8437., 14979.}	17.4576	0.0964219	newly 5 weeks cumul	71735.	56232.5	{44754., 64350.}	21.6108	0.110007	newly 64 weeks cumul	87984.	67540.5	{49364., 77478.}	23.2355	0.119784
50	newly 3 weeks spec	10269.	10816.7	{10630., 10974.}	5.33324	0.0225367	newly 3 weeks cumul	26490.	27745.6	{27488., 28043.}	4.74002	0.0200982	newly 5 weeks spec	9271.	9556.64	{9423., 9654.}	3.08101	0.0131587	newly 5 weeks cumul	46170.	47984.4	{47667., 48302.}	3.92982	0.0167348	newly 55 weeks cumul	81778.	82645.1	{82506., 82799.}	1.06026	0.00457961
